# Effects of zinc supplementation on obesity: study protocol for a randomized controlled clinical trial

**DOI:** 10.1186/s13063-016-1651-3

**Published:** 2016-11-04

**Authors:** Kumari M. Rathnayake, KDRR Silva, Ranil Jayawardena

**Affiliations:** 1 Department of Applied Nutrition, Faculty of Livestock, Fisheries & Nutrition, Wayamba University of Sri Lanka, Makandura, 60170 Sri Lanka; 2 Institute of Health and Biomedical Innovation, Faculty of Health, Queensland University of Technology, Brisbane, QLD Australia; 3 Department of Physiology, Faculty of Medicine, University of Colombo, Colombo, Sri Lanka

**Keywords:** Zinc supplementation, Obesity, Randomized clinical trial, Adults

## Abstract

**Background:**

The prevalence of obesity is escalating alarmingly worldwide, and it is now becoming a rapidly growing epidemic in developing countries. Recent studies have reported that zinc has been implicated in altered lipid markers, insulin resistance and some obesity markers. There is a lack of evidence on zinc as a potential therapeutic agent to reduce weight and improve metabolic parameters in obese adults. The present study is designed to evaluate the effects of zinc supplementation on obese adults in Sri Lanka. Furthermore, we aim to evaluate the effects of zinc supplementation on metabolic parameters in this population.

**Methods/design:**

This study will be conducted as a randomized, double-blind, placebo-controlled clinical trial for a period of 3 months at the clinical laboratory, Department of Applied Nutrition, Wayamba University of Sri Lanka to assess the efficacy of daily zinc 20 mg supplementation in obese subjects. There will be a total of 80 subjects, aged between 18–60 years, of both genders, who are obese (body mass index (BMI) ≥25). Subjects will be stratified according to age, gender and BMI and randomly assigned into the test and placebo groups in a 1:1 ratio. The treatment drug is a capsule containing elemental zinc 20 mg as the active ingredient (as zinc sulphate). The placebo capsule will contain lactose monohydrate. The subjects will receive either zinc capsules or placebo daily for 3 months. The study treatments will be double blinded to both investigator and subject. The visits and the evaluations will be as follows: screening (visit 0), baseline (visit 1) and 3 month (visit 2). The primary outcome will be weight reduction among the obese subjects. Secondary outcome measures include glycaemic status (fasting blood glucose), lipid parameters (total cholesterol, triglyceride levels, high-density lipoprotein cholesterol, low-density lipoprotein cholesterol) and blood pressure.

**Discussion:**

The trial protocol will aim to establish the effects of zinc supplementation on weight reduction and metabolic risk parameters among obese subjects.

**Trial registration:**

Sri Lanka Clinical Trials Registry: SLCTR/2014/020. Registered on 18 September 2014.

## Background

The prevalence of obesity is escalating alarmingly worldwide, and now it is becoming a rapidly growing epidemic in developing countries including south Asia [1]. Obesity is a major public health problem in Sri Lanka [2]. The level of obesity in Sri Lanka has increased several fold during last two decades, and nearly a quarter of adults have a body mass index (BMI) of more than 25 [3]. More seriously, obesity-associated metabolic problems such as diabetes (~11 %) [4], hypertension (~20 %) [4] and metabolic syndrome (~25 %) [5] are also highly prevalent among Sri Lankan adults. On the other hand, several micronutrient deficiencies are also still major public health issues in Sri Lanka, including zinc deficiency [6].

A low plasma zinc level is associated with obesity [7, 8]. Zinc may regulate serum leptin concentration [9] and appetite control. Zinc (Zn), an essential micronutrient and a component of many enzymes, is involved in the synthesis, storage and release of insulin [10]. Studies have reported that Zn has been implicated in altered lipid markers, insulin resistance, oxidative stress, inflammatory markers [11, 12], adiposity and serum leptin level [13]. Furthermore, a recent review and meta-analysis reported that zinc supplementation has favourable effects on plasma lipid parameters and that it also significantly reduces total cholesterol, low-density lipoprotein (LDL) cholesterol and triglycerides [14]. A high prevalence of coronary artery disease (hypertension, hypertriglyceridaemia and low high-density lipoprotein (HDL) cholesterol levels), diabetes and glucose intolerance was reported in populations consuming lower intakes of dietary zinc [15]. Although there is accumulating evidence on the relationship between serum zinc and human metabolism, there is a lack of evidence on Zn as a potential therapeutic agent to reduce weight and improve metabolic parameters in obese adults. Thus, this study aims to evaluate the effects of Zn supplementation on weight reduction and metabolic parameters in obese adults in Sri Lanka.

## Methods/design

### Study design

This is a randomized double-blind, placebo-controlled clinical trial.

### Setting

**Fig. 1:**
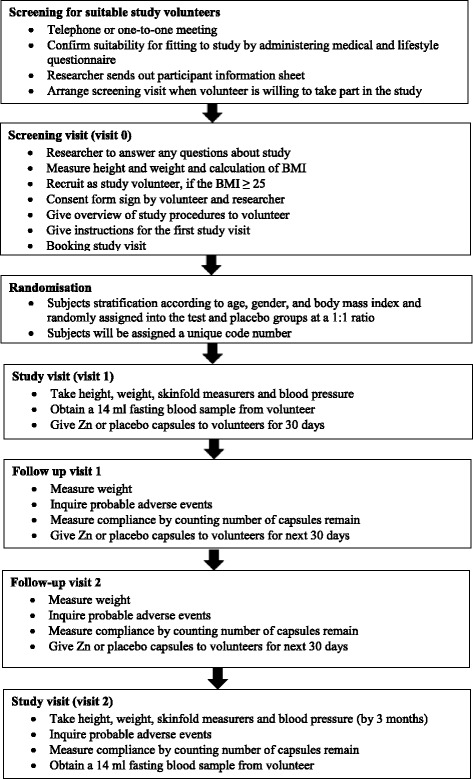
Timeline of the study

The proposed clinical trial will be held at the clinical laboratory, Department of Applied Nutrition, Wayamba University of Sri Lanka for 3 months to assess the efficacy of daily zinc 20 mg supplementation in obese subjects. Figure 1 illustrates the timeline of the study.

### Participants (inclusion and exclusion criteria)

Subjects will be obese (BMI ≥25) individuals aged between 18–60 years of both genders. Definitions for anthropometric cut-offs for obesity were based on WHO cut-offs for Asians [16]. The following exclusion criteria will be applied: subjects on any other vitamin or mineral supplementations or the current use of a weight loss medicine or special dietary modification, history of diabetes mellitus or any metabolic disease, alcohol consumption >20 g/day, presently having acute diseases or other untreated illness requiring treatment, impaired hepatic or renal functions, lactation, pregnancy or unwillingness to use an effective form of birth control for women of child-bearing years and history or presence of any condition, in the investigator’s opinion, that would endanger the individual’s safety or affect the study result. Furthermore, subjects will be suspended from the study if they demand to discontinue it and/or have low rates of compliance.

### Randomization and blinding

After the screening phase, subjects will be stratified according to age, gender and BMI and randomly assigned to one of two groups of 40 subjects each into the test and placebo. Assignment to either group will be performed by a computer-generated 1:1 randomization. The study treatments are double blinded to both investigator and subject. The supplements and placebo will be produced by Astron Lanka Limited, Ratmalana, Sri Lanka. They will be responsible for the labelling and packaging of the Zn capsule and placebo with code numbers.

### Interventions

The study will be conducted for 3 months. The visits and the evaluations will be as follows: screening (visit 0), baseline (visit 1) and 3 month (visit 2). The treatment drug is a capsule containing elemental zinc 20 mg as the active ingredient (as zinc sulphate). The supplemented Zn dose was set based on a previous study published in the literature [14]. The placebo capsule will contain lactose monohydrate. The placebo will be manufactured to have a similar appearance, shape, weight, taste and colour as the Zn capsule. The subjects will receive either one capsule of Zn or an identical placebo daily, taken after dinner for a period of 3 months. Figure 1 illustrates the schedule of the study.

### Measurements

The primary endpoint is weight reduction among obese adults. Body weight will be measured using a calibrated electronic floor scale (SECA 815 by SECA GmbH & Co. KG, Hamburg, Germany) to the nearest 0.1 kg. Height will be measured to the nearest 0.1 cm using an upright plastic portable stadiometer (IP0955, Invicta Plastics, Leicester, UK). BMI will be calculated as weight (in kilograms) divided by the square of height (in metres). Waist circumference will be measured with a non-elastic tape (SECA 203 by SECA GmbH & Co. KG, Hamburg, Germany) at a point midway between the lower border of the rib cage and the iliac crest at the end of normal expiration. Similarly, the hip circumference also will be measured at the widest part of the buttocks at the intertrochanteric level to the nearest 0.1 cm. All anthropometric measures will be taken by trained research assistants using standard equipment according to the standard guidelines [17]. In addition, glycaemic status (fasting blood glucose), lipid parameters (total cholesterol, triglyceride levels, HDL cholesterol, LDL cholesterol) and blood pressure will be measured. Glucose in the plasma will be determined by the glucose oxidase/peroxidase (GOD-Perid) method using commercially available kits (ProDia International, USA) and a chemistry analyser (Erba Chem-7, UK). Total cholesterol, triglyceride levels and HDL cholesterol (HDL-C) will be measured with enzymatic colorimetric assays using commercially available kits (ProDia International, USA) and an Erba Chem-7 chemistry analyser (Erba Molecular, Cambridgeshire, UK). LDL cholesterol (LDL C) will be estimated based on the Friedewald equation [18]. Systolic (SBP) and diastolic blood pressure (DBP) will be measured after at least a 10-min rest with Omron IA2 digital blood pressure monitors (Omron Healthcare, Singapore).

### Sample size

The minimum estimated sample size was 35 for each study arm, and a 15 % dropout rate was calculated. Finally, 40 subjects were recruited for each group.

### Dietary assessment

A 7-day diet diary will be used to measure energy and nutrient intakes and they will be calculated using NutriSurvey 2007 (EBISpro, Germany) nutrient analysis software, modified for Sri Lankan food items and recipes.

### Compliance calculation

Subjects are asked to return any remaining drugs, and their compliance will be evaluated by using the formula:

$$ \mathrm{Compliance}\left(\%\right)=\frac{\mathrm{Distributed}\ \mathrm{capsules}\hbox{-} \mathrm{remainining}\ \mathrm{capsules}}{\mathrm{Distributed}\ \mathrm{capsules}}\times 100 $$

### Data protection and confidentiality

Confidentiality will be maintained by giving all volunteers a unique identification code. Names will not be used in any reports or publications. Data will be kept for a maximum of 5 years. The files which match the personal details of the participants with their individual identification code will be held separately in a locked filing cabinet within the Department of Applied Nutrition and will be accessible only to the study investigators.

### Statistical analysis

SPSS version 16 (SPSS Inc., Chicago, IL, USA) will be used for data analysis. For categorical data, Pearson’s chi-squared test will be used to verify the association in the treatment and placebo groups. For binary and continuous outcomes, logistic and linear regression models will be used, respectively. Statistical analysis will be performed by descriptive analysis, with numerical variables expressed as the mean and standard deviation (SD) or median and interquartile range. The Kolmogorov-Smirnov test will be used to test the normality of the sample distribution. The Student *t* test will be used in cases with a normal distribution to compare means between the two groups. The Wilcoxon test will be used in cases with a non-normal distribution. *P* values <0.05 will indicate a significant association in all of the statistical tests used.

## Discussion

The level of obesity has reached epidemic proportions in many countries. Several non-communicable diseases such as diabetes, metabolic syndrome, ischaemic heart diseases and certain cancers are strongly associated with obesity. Obesity is a major health burden in south Asian countries including Sri Lanka [3]. Only a very limited number of clinical trials have been conducted in Sri Lanka, although the country is experiencing several health burdens [2, 4, 5]. Experiments on low-cost and effective treatment methods for major health disease in Sri Lanka are greatly needed.

In parallel to the problems of obesity and non-communicable diseases (NCDs), south Asian countries are facing malnutrition and micronutrient deficiencies. A systematic review and meta-analysis showed satisfactory effects of Zn supplementation on diabetes and other metabolic markers, especially studies conducted in developing countries [19]. Hashemipour et al. showed significant body weight reduction and metabolic improvement in a group of pre-pubertal obese children in Iran [11]. However, there is a scarcity of published data on zinc supplementation on obese adults from developing countries where marginal deficiencies are possible.

The proposed study, as far as we are aware, will be the only study of its type to be conducted in Sri Lanka and one of the few studies conducted elsewhere in the world. The findings will be of major importance to clinical practice for the management of obesity using nutritional supplements and to the general public, who will learn about the health impact of taking Zn supplements to lose weight and thus avoid some chronic diseases. The results will also assess mineral deficiencies in people who are obese and are at risk of developing obesity-related diseases. Because of the fundamental nature of the work proposed in this project, the findings are likely to be of significance to a wide range of users such as hospital health care teams and ministries of health who conduct supplementation and fortification programmes to improve the health status of the general public.

### Strengths and weaknesses

The first advantage is that randomized, double-blind placebo controlled trials represent the highest level of research evidence. Second, there are no reported adverse effects of a dose of 20 mg/d of zinc supplementation on otherwise healthy adults. Previous studies on supplementation with zinc (20 mg/d) did not show any adverse effects [11]. We expect a high level of patient compliance in this study. Third, the measurement of metabolic parameters such as plasma lipids, glucose and blood pressure along with anthropometric changes will provide a complete picture of the effect of zinc supplementation on obesity and associated risk factors. However, we do not have the funding or the technical facilities to measure serum zinc values, which may hinder the ability to interpret results related to patients’ zinc status.

### Trial status

This trial is in the recruitment stage.

## Abbreviations

DBP: Diastolic blood pressure
SBP: Systolic blood pressure
SLCTR: Sri Lanka Clinical Trials Registry
HDL-C: High-density lipoprotein cholesterol
LDL-C: Low-density lipoprotein cholesterol
SD: Standard deviation
NCD: Non-communicable disease
